# Impulsivity and Venturesomeness in an Adult ADHD Sample: Relation to Personality, Comorbidity, and Polygenic Risk

**DOI:** 10.3389/fpsyt.2020.557160

**Published:** 2020-12-14

**Authors:** Oliver Grimm, Heike Weber, Sarah Kittel-Schneider, Thorsten M. Kranz, Christian P. Jacob, Klaus-Peter Lesch, Andreas Reif

**Affiliations:** ^1^Department of Psychiatry, Psychosomatic Medicine and Psychotherapy, University Hospital, Goethe University, Frankfurt, Germany; ^2^Klinik für Psychiatrie und Psychotherapie der Medius Klinik, Kirchheim unter Teck, Germany; ^3^Division of Molecular Psychiatry, Center of Mental Health, University of Wuerzburg, Wuerzburg, Germany

**Keywords:** impulsivity, ADHD, polygenic risk score, venturesomeness, substance abuse disorder, attention, hyperactivity

## Abstract

While impulsivity is a basic feature of attention-deficit/hyperactivity disorder (ADHD), no study explored the effect of different components of the Impulsiveness (Imp) and Venturesomeness (Vent) scale (IV7) on psychiatric comorbidities and an ADHD polygenic risk score (PRS). We used the IV7 self-report scale in an adult ADHD sample of 903 patients, 70% suffering from additional comorbid disorders, and in a subsample of 435 genotyped patients. Venturesomeness, unlike immediate Impulsivity, is not specific to ADHD. We consequently analyzed the influence of Imp and Vent also in the context of a PRS on psychiatric comorbidities of ADHD. Vent shows a distinctly different distribution of comorbidities, e.g., less anxiety and depression. PRS showed no effect on different ADHD comorbidities, but correlated with childhood hyperactivity. In a complementary analysis using principal component analysis with *Diagnostic and Statistical Manual of Mental Disorders, Fourth Edition* ADHD criteria, revised NEO Personality Inventory, Imp, Vent, and PRS, we identified three ADHD subtypes. These are an impulsive–neurotic type, an adventurous–hyperactive type with a stronger genetic component, and an anxious–inattentive type. Our study thus suggests the importance of adventurousness and the differential consideration of impulsivity in ADHD. The genetic risk is distributed differently between these subtypes, which underlines the importance of clinically motivated subtyping. Impulsivity subtyping might give insights into the organization of comorbid disorders in ADHD and different genetic background.

## Introduction

Attention-deficit/hyperactivity disorder (ADHD) is a common neurodevelopmental disorder that manifests in early childhood and continues into adulthood. Adult ADHD is characterized by a complex pattern of comorbidities, e.g., anxiety, depression, or substance abuse disorders (1), and shows significant heritability (2, 3). Its trait-like characteristics are underscored by the fact that PRSs are related to traits, reminiscent of the disorder, in the general population (4).

During the developmental trajectory of ADHD, patients often contract comorbid disorders (2). In a study of more than 18,000 Swedish twins, presence of ADHD leads to an increase of 33% for regular nicotine use, 154% for multiple drug use, and 258% for alcohol dependence (5). Adult ADHD increases risk for anxiety disorders by 200% and major depression by 450% (6, 7). As increased impulsivity has a role in substance use disorder (SUD), it also may be a risk factor for negative outcomes in patients with adult ADHD (8, 9).

As ADHD shows a persisting course in the majority of cases, it is in some respect more comparable to a trait than a disease-specific state (10). Several studies already reported specific personality traits for ADHD; e.g., ADHD is often assumed to go along with adventurous novelty seeking, as well as highly impulsive behavior (11). In this study, we were especially interested in impulsivity, because impulsivity has some clearly delineated neurobiological roots, e.g., specific changes in the dopaminergic system and a specific genetic background. Impulsivity is discussed as a trait with a diminished prefrontal dopaminergic release and a higher striatal dopaminergic neurotransmission (12–14). This resembles theories of neurobiological mechanisms in ADHD (15, 16) discussing the prefrontal cortex and its dopaminergic modulation as a “brake” for striatal top-down control. This mechanistic link between ADHD and impulsivity is illustrated by the fact that the well-known ADHD drug methylphenidate reduces dose-dependently the pre-mature responding and normalizes the density of D_2_ receptors in rats bred for high impulsivity (17).

Impulsivity is a heterogeneous and multidimensional construct (18, 19). It has been associated with neurocognitive measures as temporal discounting or nucleus accumbens activity on a neurobiological level (14). Impulsivity includes constructs such as delay of gratification, executive control, lack of effort, and grip (14). It comprises aspects of executive (volitional), motivational (precipitated by fear or anxiety), and automatic attentional inhibition to account for this; Eysenck proposed a self-rating scale (I7) that characterizes impulsiveness and venturesomeness as two independent dimensional traits of impulsivity (20). While impulsiveness has been conceptualized as spontaneous behaving without realizing the risk in the behavior, venturesomeness is defined as being conscious of the risk but acting anyway. Several other self-rating scales of impulsivity do not distinguish these two aspects. A study that assessed the principal component structure of the Eysenck Impulsiveness Scale (20), the Dickman Impulsiveness Scale (21), Barratt's Impulsiveness Scale (22), and the Behavioral-Inhibition/Behavioral Activation scales (23) provided evidence for the components used in the Impulsiveness and Venturesomeness scale (IV7). There, impulsiveness corresponded to a dimension called “non-planning dysfunctional” in contrast to “functional venturesomeness” (23, 24). In addition to these studies underscoring the construct's validity, satisfactory reliability has been reported in the original study (α reliability = 0.79–0.84), as well as for the German translation in comparison to the English version (20, 25). However, while even laymen accept the stereotype of ADHD patients as being “thrill-seeking,” no study looked systematically at how adult ADHD and its psychiatric comorbidities cluster along these two dimensions of impulsivity.

Does studying aspects of impulsivity give us a better insight into the disease? A convincing answer would demonstrate a link between functional impairment and a personality trait related to impulsivity. Indeed, this has been shown for self-directedness (related to neuroticism and emotional-regulation), where patients with higher functional impairment have lower self-directedness independent of psychiatric comorbidity and ADHD subtype (26).

But how does the genetic risk of ADHD relate to different aspects of impulsivity? So far, there is no study comparing impulsivity (e.g., via using the IV7) and general personality traits (e.g., using NEO-PI-R) and relating them to psychiatric comorbidities and PRSs in an adult ADHD sample where stable personality traits can be assumed (as compared to children and adolescents). Both ADHD and impulsivity have been shown to have a high heritability (27), which was further underscored by a recent ADHD genome-wide association study (GWAS) (28). The latter enables the calculation and subsequent application of so-called PRSs, which are sum scores of the individual genetic risk based on whole-genome genotyping (29, 30). While previous candidate-gene studies indicated some link to impulsivity and certain ADHD-traits, these were not always replicable (3). ADHD PRSs correlate with several psychiatric and physical variables linked to ADHD, e.g., body mass index, general cognitive ability, neuroticism, and smoking (31). However, most large-scale studies do not incorporate personality traits other than neuroticism. Therefore, we ask whether an ADHD PRS can predict specific ADHD subdimensions and whether some comorbidities, e.g., SUD, show a higher PRS load. As a recent study underscored that childhood and adulthood genetic risk correlates strongly, we were interested in testing whether patients with stronger childhood symptoms had different PRS (32).

The objectives of the present study are to test whether venturesomeness and impulsivity according to the IV7 scale do indeed differ between ADHD cases and controls and to further characterize how psychiatric comorbidities cluster along different impulsivity axes in our ADHD sample. As complementary, bottom-up analysis, we want to look how a principal component analysis (PCA) divides the personality traits and ADHD symptoms of our ADHD patient sample and how this relates to comorbid disorders. Finally, we were especially interested in understanding how the analysis above relates to an ADHD PRS in a genotyped subset of our sample.

## Methods

### Subjects

Patients were suffering from an adult ADHD according to the diagnostic criteria of *Diagnostic and Statistical Manual of Mental Disorders, Fourth Edition* (*DSM-IV*): onset before the age of 7 years via retrospective diagnosis, lifelong persistence, and current diagnosis. Age at recruitment was between 18 and 65 years. Withdrawal of patients with SUD was treated in an inpatient setting. Patients with autism spectrum disorder, psychotic disorders, intelligence below IQ < 80, or any ADHD-like symptom better accounted for by other mental illness were excluded. Healthy controls were screened for the absence of psychiatric disorders. The Ethics Committee of the University of Würzburg approved the study, and written informed consent was obtained from all patients after procedures, and the aims of the study had been fully explained. In the present study, we discuss two ADHD samples: first, the complete sample (called “larger” sample) and second a subgroup (defined by the availability of PRSs, see below, called “smaller” sample).

### Clinical Assessment

Patients of the Department of Psychiatry and Psychotherapy, University of Würzburg, referred for diagnostic assessment and treatment of ADHD, were screened with the structured clinical interviews of Axis I [severe combined immunodeficiency (SCID I)] and Axis II (SCID II) disorders by trained raters. ADHD symptoms were scored as being present or not on nine *DSM-IV* inattentiveness criteria and on nine hyperactivity/impulsivity criteria based on a clinical interview. The diagnosis was based on a one-time clinical interview, which retrospectively assessed childhood symptoms. Additional information was used if available: school reports (which are done routinely for the first 2 years in German schools), information from partners, relatives or friends.

Inclusion criteria were confirmed adult ADHD according to the diagnostic criteria of *DSM-IV*, defined by onset before the age of 7 years and current diagnosis.

### Personality Traits

Traits were assessed by self-rating scales. The participants filled in the revised NEO Personality Inventory (NEO-PI-R) and the IV7 (20). The NEO-PI-R assesses the dimensions of the so-called Big Five personality traits (openness to experience, conscientiousness, extraversion, agreeableness, and neuroticism) with 240 items. The IV7 assesses the dimensions of impulsiveness (Imp), venturesomeness (Vent), and, as a control category, empathy—with 54 yes/no items. The empathy (Emp) scale was included to provide meaningful buffer items to relieve the monotony. Example for impulsivity question include, e.g., “Do you often get into a jam because you do things without thinking?” and for venturesomeness, e.g., “Would you enjoy water skiing?”

### PRS of ADHD

Genotype data were generated using the PsychChip array (15048346 B) with HumanCore, Human Exome, and custom content. Normalized intensity values were obtained using Illumina's. GenomeStudio v2010.3 with the calling algorithm/genotyping module version 1.8.4. Individuals with a call rate >95% were included in the final sample.

PRSs were computed for each of the *n* = 435 participants with available GWAS data using PRSice2 software (http://www.prsice.info/). We used the mega GWAS summary statistics of the Demontis et al. (28) study as the base dataset (28). PRS calculation was performed at standard settings (clump-kb 250, clump-kp 1.0, clump *r*^2^ 0.1, interval 5e-05, lower 5e-08, stat OR). After clumping, PRSice runs a logistic regression to find the best *p* threshold based on the explained variance (Nagelkerke's pseudo-*r*^2^ correlation). Full variance explained, *R*^2^ =.023, regression coefficient = 5656.22, threshold = 0.494, *p* = 0.000322.

Linear regression models were used to estimate associations between PRSs and six phenotypes (the IV7 scores and the three clinical components from the PCA) in the Würzburg ADHD sample. PRS were then estimated by the software tool PRSice for each target sample using all available SNPS of the meta-analysis, which were available in our sample (no *p*-value thresholding), multiplying the natural log of the odds ratio of each variant by the allele number of each variant. We controlled for population stratification by including four principal components as covariates for population stratification, regressed them out of the PRS, and used the residual for calculation of linear regression models.

### Statistical Analyses

Statistical analysis was done with SPSS 25 (33). Frequencies of comorbid Axis I disorders were calculated for the entire adult ADHD sample. Differences in the IV7 subscores (Imp, Vent and Emp) between patients and controls were tested by independent *t*-tests. Personality traits (as measured by NEO-PI-R and IV7) were compared using general linear models; age and sex were included as covariates; impulsivity, venturesomeness, and empathy were the dependent variables. For a correlation in the number of symptoms according to *DSM-IV* criteria with IV7 subscales, we used a Spearman correlation.

To get further insights into how different personality traits cluster on comorbidities in ADHD, we performed a PCA in SPSS 25. We used the unrotated solution because rotation needs *a priori* assumptions, e.g., different algorithm, different rotation criteria, and the choice of the number of components to be rotated (34). PCA with rotation according to varimax or equimax in the small and the large sample did not alter the interpretation of the data; therefore, we report only the unrotated solution. Component selection was done by extraction of all eigenvariates >1. Scree plot (see Supplementary Figure 1) and explained cumulative variance are reported.

## Results

### Demographic and Clinical Characteristics

An overview about distribution of age, sex, clinical characteristics, available genotyping, and mean numbers of personality scales can be found in (Table 1).

**Table 1:** Overview of demographics, psychopathology, available genotyping, and personality.

		**ADHD diagnosis**			
		**Healthy**		**Patients**	
		**M**	**No./SD**	**M**	**No./SD**
Age		46.85	10.21	45.00	10.46
Gender	Male		46		450
	Female		47		453
**Available genotyping (small sample)**					
Genotyped for PRS					453
Comorbid disorders					
Depression			0		482
Anxiety			0		247
Alcohol	Abuse		0		78
	Addiction		0		79
Cannabis	Abuse		0		93
	Addiction		0		128
Sedatives	Abuse		0		16
	Addiction		0		15
Stimulants	Abuse		0		53
	Addiction		0		31
Opiates	Abuse		0		22
	Addiction		0		13
Cocaine	Abuse		0		43
	Addiction		0		19
Hallucinogens	Abuse		0		42
	Addiction		0		11
**Personality questionnaires IV7 and NEO-5**					
Impulsivity		5.63	SD 3.03	10.40	SD 4.08
Venturesomeness		8.20	SD 3.25	8.10	SD 4.23
Empathy		10.71	2.63	10.99	2.83
Neuroticism		141.8	9.3	148.8	10.6
Extraversion		144.9	8.6	150.9	10.3
Openness		151.8	9.4	153.1	10.2
Agreeableness		151.2	9.8	154.2	10.8
Conscientiousness		151.4	9.9	153.7	10.5

*Table 1 shows age and gender distribution in two columns for healthy controls and ADHD patients. It gives the number of genotyped patients, which forms our smaller sample used in separate analysis. It lists comorbid disorders. Grading between “abuse” and “addiction” was available for substance use disorders. The section on personality measures gives the mean for the IV7 and the NEO. No., number; M, mean; SD, standard deviation*.

### Correlation Between IV7 Scales and Clinical Characteristics

We looked at the relationship between impulsivity, venturesomeness, and empathy with inattention and hyperactivity in both adulthood and childhood with a Spearman correlation. Impulsivity was significantly correlated with hyperactivity and inattention in adulthood and childhood (*p* < 0.001, *r* > 0.29), with the correlation between impulsivity and childhood hyperactivity showing the highest correlation (*r* = 0.46, *p* < 0.001). Venturesomeness was strongly correlated with childhood hyperactivity and inattention (*p* < 0.001, *r* > 0.14), but not adulthood. Empathy correlated with adulthood symptoms (*p* < 0.001, *r* > 0.11) but not childhood symptoms.

### Differences in the IV7 Scores Between Controls and ADHD

In an independent *t*-test, we compared the complete sample (identical with the large sample without PRS) with our healthy controls: only impulsiveness (*p* < 0.001, *df* = 1,994, *t* = 10.97, *d* = 1.32), but not venturesomeness (*p* = 0.82, *df* = 1,994, *t* = 0.22, *d* = 0.02) and empathy (*p* = 0.36, *df* = 1,994, *t* = 0.91, *d* = 0.1), was different between ADHD cases and controls.

### Receiver Operating Characteristic Curve for Personality Traits in Differentiating ADHD

In comparison to all other personality dimensions, impulsiveness was best in predicting ADHD [area under the curve (AUC) = 81.7%, *p* < 0.001], followed by extraversion (AUC = 66.9%, *p* < 0.001) and neuroticism (AUC = 69.7%, *p* < 0.001). Therefore, impulsiveness gives good testing characteristics; neuroticism or extraversion performs poorly [according to the commonly used test criteria (35)]. When impulsiveness, neuroticism, and extraversion are combined, the result is only slightly better than impulsiveness alone (AUC = 83.2%, *p* < 0.001), meaning that no additional value is gained by using a sum index across the best three traits.

### Linear Regression Analysis of IV7 Subscales and Comorbid Disorders

We calculated three linear regression models with impulsiveness (Imp), venturesomeness (Vent), empathy (Emp) with and without PRS, see Table 2. While our analysis focused on Imp and Vent, we included Emp as a secondary endpoint. First, it can demonstrate that questions from the same questionnaire are completely unrelated to any impulsivity-based construct, and second, previous research has shown lowered empathy in adults with ADHD and SUDs (36). Therefore, we were interested in testing empathy as a way of characterizing a subset of our sample. As we calculated separate models with and without the PRS, here referred to as the smaller sample (*n* = 435) with PRS and the larger sample without PRS (*n* = 903). All other covariates were the same between the four models.

**Table 2:** Regression models IV7 and PRS.

**Variables**	**Impulsivity**			**Venturesomeness**			**Empathy**		
	**Standard coefficient**	***T***	**Sig**.	**Standard coefficient**	***T***	**Sig**.	**Standard coefficient**	***T***	**Sig**.
	**β**			**β**			**β**		
**Larger sample with covariate PRS**, ***n*** **=** **903**									
Constant		14.178	<0.001		21.323	<0.001		13.582	<0.001
Sex	0.072	2.081	0.038	−0.293	−9.387	<0.001	0.342	10.305	<0.001
Age	−0.112	−3.118	0.002	−0.189	−5.857	<0.001	0.083	2.430	0.015
Depr	0.003	0.075	0.941	−0.053	−1.687	0.092	0.043	1.290	0.197
AnxDis	0.007	0.190	0.849	−0.140	−4.460	<0.001	−0.004	−0.106	0.915
SUDAlc	0.086	2.259	0.024	0.022	0.638	0.524	−0.003	−0.069	0.945
SUDCan	0.085	1.994	0.047	0.135	3.514	<0.001	0.055	1.356	0.175
SUDSed	0.007	0.207	0.836	0.041	1.266	0.206	0.003	0.073	0.942
SUDSti	0.032	0.708	0.479	−0.010	−0.260	0.795	0.065	1.511	0.131
SUDOpi	0.071	1.856	0.064	0.035	1.027	0.305	0.012	0.327	0.744
SUDCoc	0.020	0.467	0.640	0.050	1.297	0.195	−0.110	−2.698	0.007
SUDHal	0.016	0.378	0.705	0.011	0.290	0.772	−0.013	−0.303	0.762
**Smaller sample with covariate PRS**, ***n*** **=** **435**									
Constant		5.518	<0.001		7.976	<0.001		4.213	<0.001
Sex	0.188	3.805	<0.001	−0.242	−5.302	<0.001	0.361	7.728	<0.001
Age	−0.108	−2.142	0.033	−0.208	−4.501	<0.001	0.091	1.928	0.055
PRS	0.045	0.935	0.351	0.016	0.373	0.709	−0.024	−0.531	0.596
Depr	−0.016	−0.329	0.743	−0.086	−1.901	0.058	0.074	1.611	0.108
AnxDis	0.039	0.796	0.427	−0.164	−3.642	<0.001	0.023	0.491	0.624
SUDAlc	0.143	2.582	0.010	−0.006	−0.125	0.900	−0.048	−0.928	0.354
SUDCan	−0.028	−0.491	0.624	0.143	2.666	0.008	−0.020	−0.366	0.715
SUDSed	0.008	0.152	0.879	0.051	1.065	0.287	0.051	1.024	0.307
SUDSti	0.070	1.153	0.250	−0.011	−0.196	0.845	0.140	2.441	0.015
SUDOpi	0.048	0.932	0.352	0.041	0.877	0.381	0.042	0.858	0.391
SUDCoc	−0.069	−1.119	0.264	0.059	1.039	0.299	−0.106	−1.813	0.071
SUDHal	0.147	2.377	0.018	0.017	0.295	0.768	−0.026	−0.439	0.661

*Table 2 shows the adjusted β values. T effect-sizes and p-values for the linear regression models with clinical comorbidities as covariates. SUD, substance use disorder; AnxDis, anxiety disorder; Depr, major depression; Alc, alcohol; Can, cannabis; Sed, sedatives; Opi, opioids; Coc, cocaine; Hal, hallucinogens; PRS, polygenic risk score*.

Patients with alcohol and hallucinogen abuse or dependency showed higher impulsiveness scores. No other diagnostic entities or the PRS were significant for the dependent variable impulsiveness. Patients with anxiety disorder diagnosis showed significantly lower venturesomeness scores. Patients with cannabis abuse or dependency showed higher venturesomeness scores. No other diagnostic entity was significant for the dependent variable venturesomeness.

Patients suffering from stimulant abuse or dependency disorder showed higher scores on empathy in the smaller sample with PRS covariate, but not in the complete sample, pointing to a false-positive effect (see Table 2).

As all regression models found strong effects for a decline of venturesomeness and impulsiveness with age and lower score for females, we looked in additional analyses whether there are significant interactions between IV7 scales and age or sex. No interaction term in scale ^*^ age or scale ^*^ sex was significant (*p* > 0.24).

### PCA of Personality, Inattentiveness, and Hyperactivity and PRS

We combined the dimensional scales of the PRS (only smaller sample), NEO-PI-R, the IV7, and the ADHD inattentive and hyperactivity scale in a PCA to gain further insight how adult ADHD can be differentiated into different (orthogonal) components (Table 3). Three components explained 59.34% of the variance; in the smaller sample within PRS, three components explained 51.71% of the variance. In a complementary analysis of the regression models above, we used these components as dependent variables and tried to predict them with the comorbid diagnosis. The results below are reported for the smaller sample unless otherwise stated.

**Table 3:** Principal component analysis correlation table.

	**Component matrix**					
	**Components sample**, ***n*** **=** **435 with PRS**			**Components sample**, ***n*** **=** **903 without PRS**		
	**1**	**2**	**3**	**1**	**2**	**3**
Impulsivity	0.396	0.613	0.375	0.400	0.685	0.273
Venturesomeness	0.260	0.573	−0.427	0.230	0.647	−0.441
Empathy	0.288	−0.386	0.541	0.291	−0.311	0.569
PRS	−0.009	0.356	0.044	–	–	–
Inattention	0.115	0.046	0.685	0.113	0.094	0.719
Hyperactivity	0.377	0.454	0.212	0.322	0.416	0.267
Neuroticism	0.608	−0.282	0.217	0.655	−0.262	0.136
Extraversion	0.822	0.114	−0.006	0.810	0.136	−0.015
Openness	0.749	−0.140	−0.201	0.767	−0.159	−0.185
Agreeableness	0.798	−0.087	−0.169	0.818	−0.062	−0.121
Conscientiousness	0.625	−0.323	−0.308	0.667	−0.365	−0.266

*Table 3 shows the loading of the three non-rotated component solution of the PCA. The first three left columns belong to the solution with the smaller but genotyped sample; therefore, it includes the polygenic risk score (PRS). There is no qualitative change in component loading for all variables except PRS*.

Component 1 was predicted by SUD alcohol (*p* = 0.047, *df* = 11,421, β = 0.11, *T* = 1.997) and negatively by SUD cocaine (*p* = 0.047, *df* = 11,421, β = −0.12, *T* = −1.9), while component 2 was negatively associated with depression in our regression model (*p* = 0.004, *df* = 11,421, β = −0.19, *T* = −2.85), thus predicting resilience against depression. In the larger sample, component 1 showed in addition a significant association with depression (*p* = 0.044, *df* = 11,809, *upβ* = 0.073, *T* = 2.02). Component 2 was significantly associated with hallucinogen use (*p* = 0.028, *df* = 11,421, β = 0.12, *T* = 2.2) and negatively associated with age (*p* = 0.001, *df* = 11,421, β = −0.15, *T* = −3.23). Component 2 therefore goes together with being younger, non-depressed, risk-taking in the form of hallucinogen use, and with high impulsiveness and venturesomeness. Interestingly, there is no direct correlation with neuroticism (see Table 3, “Component matrix”).

Component 3 was associated with sex (*p* < 0.001, *df* = 11,421, β = 0.36, *T* = 7.7) representing therefore a predominantly female, inattentive dimension of ADHD with less genetic load. Interestingly, in the larger sample, the link to affective and anxiety disorder was much stronger. Here, component 3 was associated with anxiety (*p* = 0.027, *df* = 11,809, β = 1.16, *T* = 2.21) and depressive disorder (*p* = 0.03, *df* = 11,809, β = 1.43, *T* = 2.17). Thus, in the larger sample, component 3 seems to be associated with the anxious dimension of ADHD comorbidity.

To better understand the relation of the PRS to ADHD symptoms in our samples, we correlated the number of fulfilled diagnostic criteria according to *DSM-IV* for inattention and hyperactivity separately for childhood and adulthood with the PRS. Notably, neither inattention scores correlated with PRS; hyperactivity in adulthood was trend wise correlated with PRS (*p* = 0.058), and only childhood hyperactivity showed a significant correlation with ADHD PRS (*p* = 0.005).

## Discussion

Our study provided evidence that impulsivity and venturesomeness give rise to a very specific pattern of psychiatric comorbid disorders in ADHD and that not all ADHD patients are per definition “thrill-seeking.” Indeed, in a subgroup of our patients, venturesomeness seems to be even decreased as it is negatively correlated with anxiety disorders, and our sample had a high number of ADHD patients suffering from anxiety disorders. This is in line with studies of adult ADHD patients showing higher harm avoidance as personality trait or higher prevalence of anxiety disorders (6, 37). Patients with anxiety disorders will obviously try to avoid planned thrill-seeking risk behavior but can nevertheless suffer from high impulsivity.

Impulsivity has been conceptualized as a core ADHD symptom, but different aspects of impulsivity might cluster differently on comorbid disorders in ADHD (14), e.g., more Impulsivity in SUD, less venturesomeness in anxiety disorder. As impulsivities multifaceted nature might stem from different neurobiological mechanisms, this subtyping approach might even carry therapeutic implications. Impulsivity has not only been shown to have genome-wide significant hits, but a recent meta-analysis of ADHD with first genome-wide significant results allows us to calculate a subject-specific ADHD risk score for ADHD and look at how this clusters in our sample with comorbid disorders. A strong genetic correlation has been already shown for the general risk trait neuroticism and for psychiatric disorders such as smoking or major depressive disorder (28). We were especially interested in looking at different SUDs because the drugs of abuse recruit very different psychopharmacological pathways ranging from the glutamatergic system in alcohol and the endocannabinoid system in THC to dopamine in cocaine dependency; therefore, it is plausible to assume that different ADHD subtypes might show different preference to certain drugs. However, a twin study points to a general factor non-specific to drug classes (38).

Previous studies rarely used impulsivity subtypes for getting insights into substructures of comorbid disorders: In a recent study with 209 participants (72 ADHD patients), the authors studied how components of the UPPS (urgency pre-mediation perseverance sensation seeking scale) differentiate between controls and cases and psychiatric comorbidities (39). Lack of perseverance showed the strongest association with ADHD (AUC = 95%), and patients with more frequent substance abuse problems scored higher on the urgency and sensation-seeking dimensions of impulsivity. However, this study cannot readily relate to ours because of the very different impulsivity measures used. The UPPS has no clear equivalent to the venturesomeness scale of the IV7. Substance abuse is an important comorbidity in persistent ADHD (2, 40). None of these studies had a look at polygenic risk. In our sample, the relation between the IV7 and SUD was not as straightforward but depended highly on the specific drug abused. This detailed assessment, graded in abuse vs. dependency, is a clear strength of our study in comparison to studies with a more simplistic binary categorization, e.g., alcohol vs. polydrug abuse.

**Figure 1:**
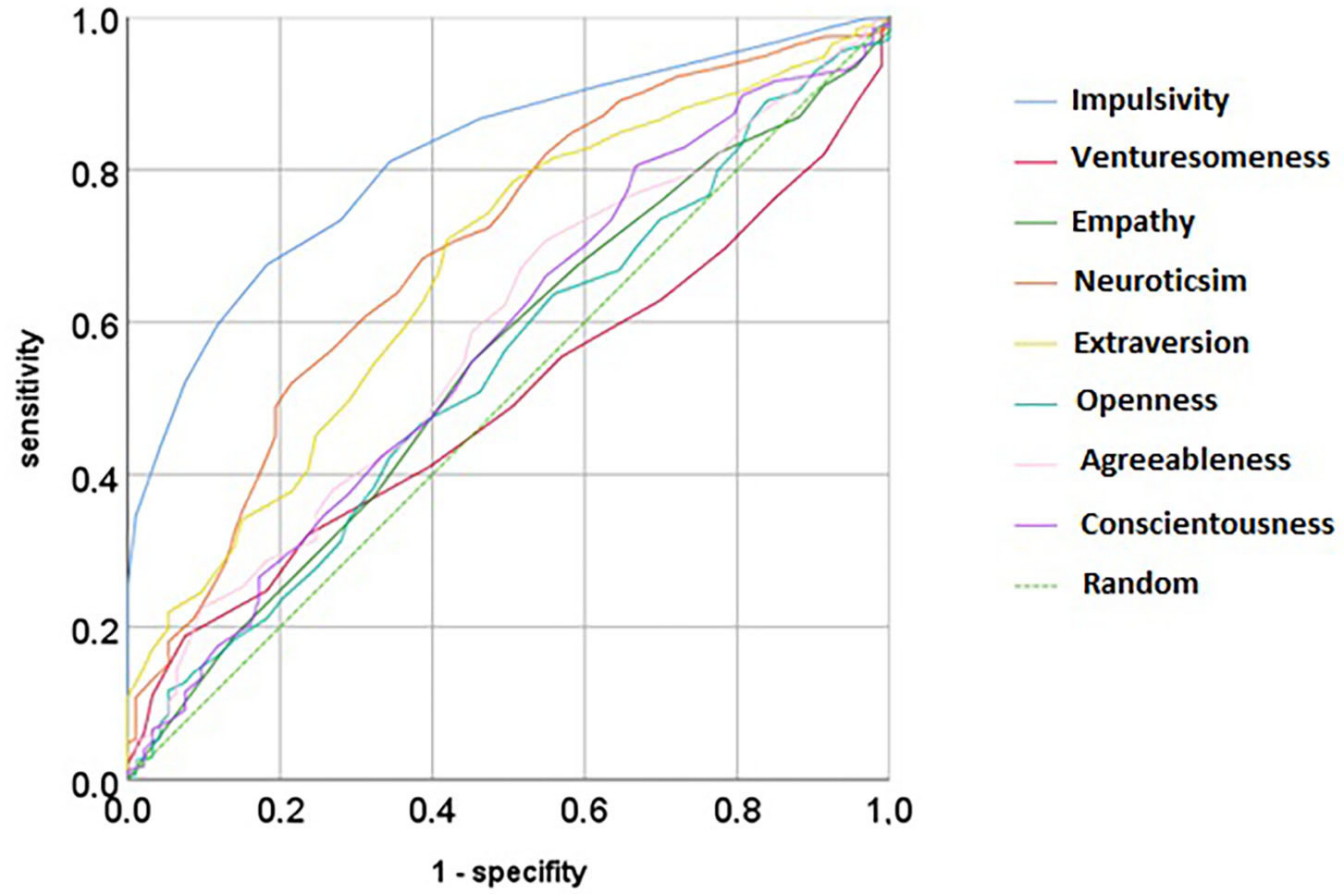
Receiver operating curve for different personality traits from the IV7 and the NEO-PI in predicting ADHD cases.

Our basic assumption that impulsiveness and venturesomeness indicate a higher level of SUDs was not met. Impulsiveness was significantly associated with alcohol or hallucinogen abuse and disorder but no other substance. Venturesomeness differentiated better between comorbid disorders. It was negatively associated with anxiety and positively with cannabis SUD. Venturesomeness bears some relation to novelty seeking or sensation seeking, a conscious risk-taking behavior. While several previous studies found indeed that adult ADHD patients have higher novelty seeking, this does not necessarily contradict our findings. In our sample, “openness to experience” or “extraversion” in the NEO-PI-R was not able to distinguish sufficiently between controls and cases, see Figure 1 and *Receiver Operating Characteristic Curve for Personality Traits in Differentiating ADHD*. Faraone et al. (37) found higher novelty seeking in the Temperament and Character Inventory in adult and subthreshold ADHD patients, indicating that adult ADHD patients are more quick-tempered, curious, impulsive, disorderly, or extravagant. However, this definition indeed mixes momentary Impulsiveness (e.g., quick-tempered) and more planned aspects (e.g., being extravagant). Additionally, our analysis was done in a larger sample, in a different cultural setting, and older patients. Venturesomeness and impulsivity were strongly negatively correlated with age. Consequently, different age groups in different studies might lead to different results.

We were especially interested in how our top-down subtyping via impulsivity and venturesomeness as well as our bottom-up subtyping via three principal components correlated with the ADHD PRS. ADHD PRSs were not able to delineate ADHD comorbid disorders and were not related to impulsivity or venturesomeness. A low prediction of personality traits by ADHD PRS in our sample does not rule out genetic effects, likely due to the relatively small sample size. However, much better power has been demonstrated in diseases with more pronounced major allele effects major gene effects, usually in the major histocompatibility complex (MHC), which depart from normally distributed quantitative PRS model, e.g., Crohn disease. In psychiatric diseases, however, the contribution of several small-scale effects might lead to low predictive power in smaller samples as ours (41). While PRS showed their strength in some ADHD studies, e.g., a recent study using PRS to predict clinically relevant phenotypes found a positive association with body mass index, depression, anxiety, alcohol intake and dependency, and neuroticism (31), this study was done in the UK Biobank sample and does not necessarily translate to ADHD samples with comorbid disorders.

Is has been a matter of debate whether childhood ADHD represents on a genetic level the same as adulthood ADHD. In previous studies, the genetic correlation between ADHD in childhood and adulthood has been estimated to be about 80% (32, 42). Interestingly, our sample showed only a significant correlation between childhood hyperactivity and the PRS. That might come from the fact that childhood ADHD cases are overrepresented in ADHD GWAS, and their symptom profile is often driven by hyperactivity.

While our sample had the advantages of thorough clinical assessment, we nevertheless want to point out some general limitations. One might argue that looking at impulsivity in a disease entity that is defined and operationalized by impulsivity is tautological. This is reflected by the high correlation between symptoms and impulsivity. Interestingly, the correlation in adulthood between venturesomeness and the number of ADHD symptoms after *DSM-IV* was not correlated, but between ADHD symptoms in childhood and venturesomeness. Finally, the more fine-grained items of the self-report questionnaire capture a different aspect of impulsivity than the broader criteria of *DSM-IV* hyperactivity in ADHD in clinical situations.

While regression models of the dependent variables venturesomeness and impulsivity give interesting insights, we looked for a PCA as a complementary data-driven method. This has the obvious advantage that the upcoming components are not correlated (as most personality measures like impulsivity are) and therefore are easier to interpret. The results of our PCA show that component 2 with high venturesomeness is different from the basic (=mean) ADHD component 1 and the more anxiety- and inattentiveness-related component 3. This is reminiscent of a recent debate based mainly on child and adolescent data whether the hyperactivity subtype with its higher comorbidity with conduct disorder can be regarded a separate entity (43–46). This ADHD–conduct disorder link fits well with the higher correlation of the venturesomeness component 2 with hallucinogen abuse and dependency, which is a marker for social activities in disregard of standard legal rules. The tertiary referral center at the University Hospital Würzburg is situated in the federal state of Bavaria, Germany, with a conservative drug policy. This fits well with our observation that venturesomeness but not impulsivity is linked to cannabis abuse. Drug policy provides a threshold for certain behaviors (e.g., cannabis or hallucinogen use), which lead to a selection of users with a conscious risk-taking profile, also known as higher venturesomeness (47).

Our PCA bears an interesting resemblance to the subtyping of combined (component 1), inattentive (component 3), and impulsive/hyperactive (component 2) subtyping and its relation to psychiatric comorbidity. However, the strong correlation of neuroticism that can be interpreted as emotional regulation ability and Impulsiveness in distinction from venturesomeness (which bears some resemblance to emotional regulation skills, too) points to the neglected issue of including emotional dysregulation as a core ADHD trait. As PCA is a purely data-driven method that leads to independent components, one might argue that it gives a better estimate of the range of possible subtypes. Therefore, we do not interpret these components as natural kinds, but they point to dimensions mirror subtypes and point strongly to the influence of venturesomeness in subtyping. While the current therapeutic and diagnostic procedures have the advantage of standardization and operationalization, they do not reflect the wide phenotypic range of ADHD in adults. As our PCA components are only “virtual” measures, they cannot solve this problem, but the usage of the IV7 might add useful additional information about subtypes.

While the regression analysis did not give significant results for PRS in relation to clinical phenotype, the PCA indicates a certain genetic influence for component 2. As this component is more abundant in young, adventurous male patients with cannabis dependence, the clinical phenotype may be closer to the core symptoms of childhood ADHD, where the sex ratio is skewed toward males, and the hyperactive component is more pronounced. As the mega-analysis GWASs include both childhood and adult cases, this might lead to a higher genetic background in component 2. However, Demontis et al. (28) found a high genetic correlation between adult and childhood ADHD. On the other side, a study using the Avon Longitudinal Study of Parents and Children (ALSPAC) sample found a higher correlation with ADHD-like hyperactivity than for inattentiveness in a young normal population sample (48).

One might question whether self-reports of impulsivity give us an insight into neurobiological mechanisms. There is conflicting insight how the dopaminergic system is related to different facets of impulsivity: While a positron emission tomography study of D_2_/D_3_ receptor binding found binding in people with high venturesomeness but no effect of impulsiveness (49), another study found higher limbic availability of the D_2_/D_3_ receptor in participants scoring high on non-functional impulsivity (comparable to Impulsiveness) (50). The UPPS scale is linked to a specific change of midbrain dopaminergic projections to the mid–cingulate gyrus during a dopaminergic challenge (51). Therefore, an easy-to-apply questionnaire such as the IV7 could be a proxy for a neurobiological state, e.g., dopaminergic responsiveness. Such an application would need longitudinal clinical studies but has some support in translational animal studies (52). A recent study in an adolescent sample indicates that body mass index is predicted by an ADHD PRS and that this effect is mediated by a monetary incentive delay task, which can be seen as a proxy for dopaminergic modulation of the reward system (53). Our PCA indicates that only in a subtype (component 2) of ADHD, the genetic background plays a role and is highly correlated to impulsivity and venturesomeness.

While our PCA analysis gives subtypes that can easily be aligned to clinical categories, subtyping has received general criticism as subtypes do not seem to be stable across time (54). This subtype instability questions research on subtypes of the disorder because even prominent differences do not seem to show future consequences. Some of these subtype instabilities might stem from diagnostic thresholds which suddenly switch individuals from belonging to one category into another, while the dimensional variable behind it (e.g., symptom count) did not change decisively. A way to improve subtype stability may be to develop a separate dimensional measure for subtyping as done in our three-component PCA. While this gives interesting insights like the venturesomeness component 2 goes together with depression resilience, it has limitations because these PCA components are “virtual” measures with no corresponding questionnaire. Without access to the same questionnaires, these results are not easily translated to other studies.

First, we showed that venturesomeness, unlike immediate Impulsivity, is not specific to ADHD. Therefore, a differentiated consideration of impulsivity in ADHD is needed. We consequently analyzed the influence of Imp and Vent also in the context of a PRS on psychiatric comorbidities of ADHD. Vent shows a distinctly different distribution of comorbidities, e.g., less anxiety and depression, more cannabis dependence. PRSs showed no effect on different ADHD comorbidities but was significantly correlated with childhood hyperactivity. In a complementary bottom-up analysis using PCA with particular emphasis on NEO-PI-R, Imp, Vent, and PRS, we identified three ADHD subtypes that had similarities and differences to conventional subtypes. These are an impulsive, neurotic type; an adventurous, less depressive, hyperactive, predominantly male type with a stronger genetic component; and a more female, inattentive type. Our study thus suggests the importance of adventurousness and the differential consideration of impulsivity in ADHD. The genetic risk seems to be distributed differently between these subtypes, which underlines the sense of clinically motivated subtyping. From a clinical point of view, this suggests that the IV7 questionnaire is a relatively easy-to-administer questionnaire that can predict affective and substance abuse–related comorbidities. As SUD, hyperactivity, amd higher genetic loading with an ADHD PRS, as opposed to higher anxiety, seem to lie on the other side of a venturesomeness continuum, clinical studies should look at the usefulness of venturesomeness in making a treatment decision and applying different therapeutic strategies, maybe even on a psychopharmacological level. On a genetic level, our data-driven component analysis suggests that adult ADSHD subtypes show distinct genetic loading of ADHD PRS. Adult ADHD is not a homogenous entity. Impulsivity subtyping might give insights into the axis on which comorbid disorders cluster. These subtypes might show a different genetic background. Future studies might use this information for therapeutic and diagnostic predictions.

## Data Availability Statement

The data analyzed in this study is subject to the following licenses/restrictions: Pseudonymized data can only be shared if patients give their consent. Requests to access these datasets should be directed to Oliver Grimm, oliver.grimm@kgu.de.

## Ethics Statement

The studies involving human participants were reviewed and approved by Ethik-Kommission der Universität Würzburg Institut für Pharmakologie und Toxikologie Versbacher Str. 9 97078 Würzburg, Germany. The patients/participants provided their written informed consent to participate in this study.

## Author Contributions

OG analyzed the data and wrote the manuscript. TK calculated PRSs. HW curated data, especially genetics data and supervised biomaterial. SK-S, CJ, K-PL, and AR collected data and biomaterial and recruited patients. All authors contributed to the manuscript.

## Conflict of Interest

AR received personal fees from MEDICE Arzneimittel Pütter GmbH & Co. KG, Shire PLC, Neuraxpharm Arzneimittel GmbH, Janssen-Cilag GmbH, Takeda (former Shire) and Servier Deutschland GmbH at present or during 36 months prior to publication. OG received personal fees from MEDICE Arzneimittel Pütter GmbH & Co. KG. SK-S received personal fees from MEDICE Arzneimittel Pütter GmbH & Co. KG and Takeda (former Shire) at present or during 36 months prior to publication. The remaining authors declare that the research was conducted in the absence of any commercial or financial relationships that could be construed as a potential conflict of interest.
