# Supplementary figures and images for: Impulsivity and Venturesomeness in an Adult ADHD Sample: Relation to Personality, Comorbidity, and Polygenic Risk
Source: Front Psychiatry. 2020 Dec 14;11:557160. doi: 10.3389/fpsyt.2020.557160 (PMC7768074; doi:10.3389/fpsyt.2020.557160)

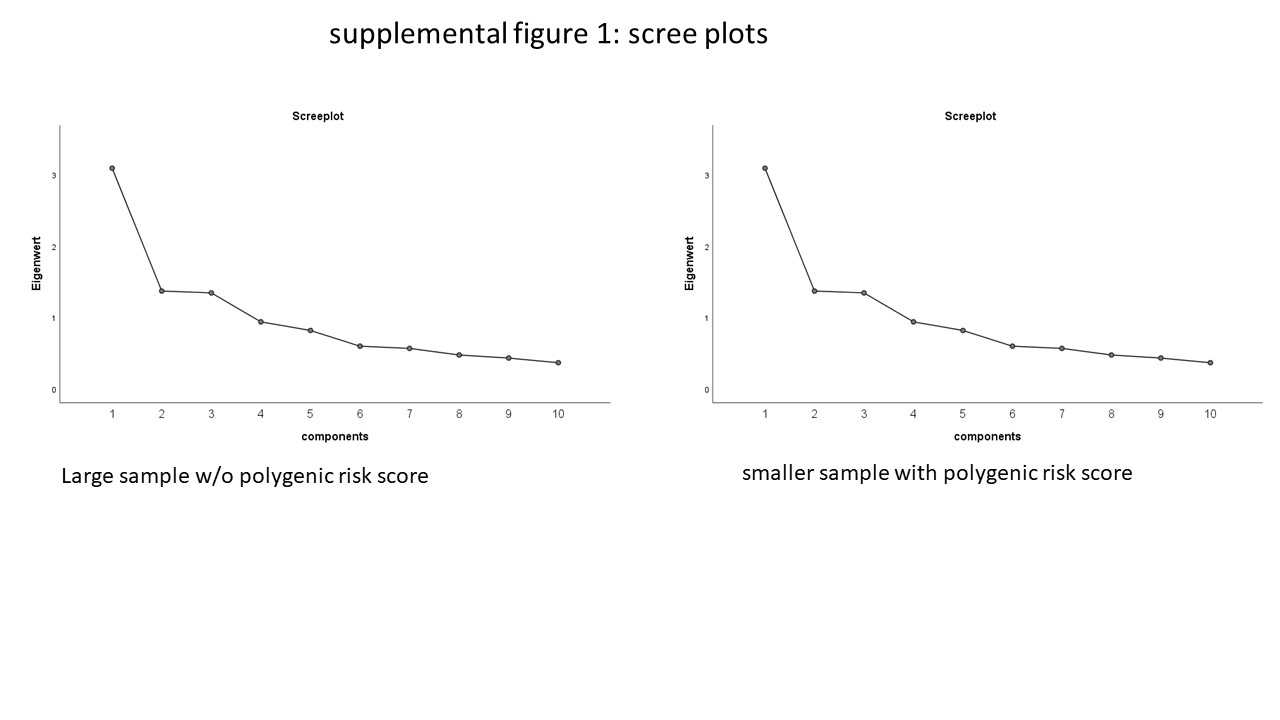

Supplement: Supplementary file 1 [file Image_1.JPEG]
